# Double Mutation of *Days to Heading 2* and *CONSTANS 3* Improves Agronomic Performance of *Japonica* Rice under Short Daylight Conditions in Southern China

**DOI:** 10.3390/ijms24087346

**Published:** 2023-04-16

**Authors:** Hongmei Wang, Yue Zhu, Linlin Wang, Chujian Xiao, Jianming Yuan, Yao-Guang Liu, Qunyu Zhang

**Affiliations:** Guangdong Laboratory for Lingnan Modern Agriculture, College of Life Sciences, South China Agricultural University, Guangzhou 510642, China

**Keywords:** *indica* to *japonica*, *DTH2*, *OsCO3*, heading and yield

## Abstract

Some progress has been made in understanding the pathways related to rice heading, but their applications to breeding *japonica* rice varieties adapted to grow in low-latitude areas (“*indica* to *japonica*”) are limited. We edited eight adaptation-related genes via a lab-established CRISPR/Cas9 system in a *japonica* variety, Shennong265 (SN265). All T_0_ plants and their progeny bearing random mutation permutations were planted in southern China and screened for changes in heading date. We found that the double mutant of *Days to heading* 2 (*DTH2*) and *CONSTANS 3* (*OsCO3*) (*dth2*-*osco3*), two *CONSTANS-like* (*COL*) genes, showed significantly delayed heading under both short-day (SD) and long-day (LD) conditions in Guangzhou and manifested great yield increase under SD conditions. We further demonstrated that the heading-related *Hd3a*-*OsMADS14* pathway was down-regulated in the *dth2*-*osco3* mutant lines. The editing of the *COL* genes *DTH2* and *OsCO3* greatly improves the agronomic performance of *japonica* rice in Southern China.

## 1. Introduction

Rice (*Oryza sativa* L.) is the staple crop for more than one-fifth of the world’s population in the world, which stresses the importance of enhancing its productivity and yield for food security. *Indica* and *japonica* are two subspecies of Asian cultivated rice that originated in Asia and have been grown worldwide. The grain quality of *japonica* varieties is superior to that of *indica* varieties, and thus *japonica* rice dominates the market in China. To meet demand, “*indica* to *japonica*” projects have been proposed for the cultivation of *japonica* rice in Southern China [1]. Traditionally, *japonica* rice is mainly grown in the temperate zones of the mid-latitudes, whereas *indica* rice is distributed in the tropics and subtropics [2]. Elite *japonica* varieties are vulnerable to short photoperiods and high temperature when growing in low-latitude areas, which greatly shortens their vegetative growth period and culminates in yield reduction and quality degradation [3,4].

Heading Date is an important agronomic trait reflecting regional adaptability and yield potential of rice cultivars. At the molecular level, rice heading date is regulated by a complicated regulatory network involving a large number of genes [5]. The relevant signaling pathways have been extensively investigated, and a few flowering time genes have been cloned, such as a *CONSTANS-like* (*COL*) gene *Heading date 1* (*Hd1*) and *Early heading date 1* (*Ehd1*) [6,7]. *Hd1* is an important regulator of photoperiod regulation of heading in rice. Early studies have shown that *Hd1* has a dual function, promoting heading under short-day (SD) conditions and delaying heading under long-day (LD) conditions [8]. *Ehd1* is a unique B-type response regulator in rice, which plays a role in promoting heading independently of *Hd1* under SD conditions [9].

*Heading date 3a* (*Hd3a*) and *Rice Flowering Locus T 1* (*RFT1*) are two florigen genes in rice, acting downstream of most flowering regulators, for example, *Hd1, Ehd1,* and several *COL* genes [10,11]. Under SD conditions, the expression of *Hd3a* is induced by activated *Hd1* and *Ehd1* and repressed by a *COL* gene, *OsCO3* [12]. Under LD conditions, *Hd3a* is down-regulated by *Hd1* but up-regulated by a *COL* gene, *DTH2*. *DTH2* and *Ehd1* also up-regulate *RFT1* [9,13], the major flowering promoter gene under LD conditions.

Despite the advances in molecular dissection of flowering pathways, there are few studies on the application of these molecular findings to the “*indica* to *japonica*” projects. We previously identified adaptation-related genes associated with the heading date in SN265, growing in northern and southern China [14,15]. In this study, we selected the eight most heading-relevant genes and aimed to identify the mutation permutations that may improve the agronomic performance of SN265 grown in Guangzhou (southern China). A lab-established CRISPR/Cas9 multiplex genome editing system was applied to generate mutation permutations [16], followed by agronomic trait evaluation. We found that the double mutation of *DTH2* and *OsCO3* (*dth2*-*osco3*), two *COL* genes [12,13], significantly delayed heading under both SD and LD conditions in Guangzhou and gained remarkable yield increase under SD conditions. Overall, these findings provide new resources for the “*indica* to *japonica*” breeding programs in South China and improve our understanding of the molecular mechanisms supporting agronomic performance in rice.

## 2. Results

### 2.1. dth2-osco3 Delays Heading in Japonica

**Figure 1:**
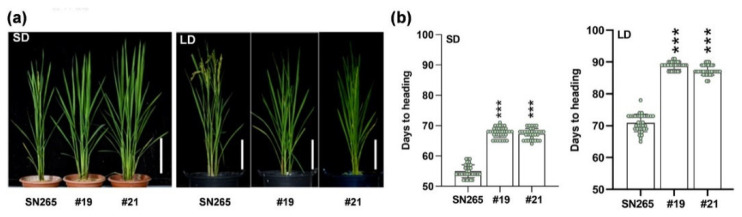
Double mutation of *DTH2* and *OSCO3* delayed heading of *japonica* rice under both SD and LD conditions. (**a**) Phenotypes of 60-d-old (the left panel) and 89-d-old (the right panel) plants of SN265 (wild type, *japonica* rice) and the double mutant *dth2*-*osco3* (two homozygous T_2_ lines #19 and #21) growing under SD conditions (the left panel) and LD conditions (the right panel). Scale bars, 20 cm (**b**) Days to heading of SN265 and *dth2*-*osco3* lines under SD (the left panel) and LD (the right panel). Error bars represent SD of three biological replicates. Values are means ± SD. ***, *p* < 0.001.

Selecting appropriate allele combinations facilitates breeding programs targeting broad adaptation. Here, we aim to improve the yield potential of *japonica* varieties growing in South China by pinpointing beneficial mutation permutations conferring delayed heading in Guangzhou, a subtropical region. The eight most heading-relevant genes, *DTH2*, *OsCO3*, *LATE-FLOWERING 1* (*OsLF1*), *SET DOMAIN GROUP PROTEIN* 725 (*SDG725*), *GLUTAMINE SYNTHETASE 1*(*OsGS1*), *Dof zinc factor 12* (*OsDof12*), *Nuclear factor Y C2 subunit* (*OsNF-YC2*), and *PURINE PERMEASE 7* (*OsPUP7*), were selected for editing from the genes differentiated between *japonica* and *indica*, which were identified in a previous genome-wide study [15]. The CRISPR/Cas9-based multiplex gene editing vectors containing the gRNAs of these genes were introduced into SN265, a *japonica* cultivar, via Agrobacterium-mediated transformation. Among all the homozygous T_2_ lines obtained, two lines, #19 and #21, carrying double mutations of *DTH2* and *OsCO3,* showed a significant decrease in days to heading (DTH) under both SD and LD conditions (Figure 1a). Single-base insertions were observed in the coding regions of both *DTH2* and *OsCO3* in both #19 and #21, resulting in frameshift mutations (Figure S1a,b). Under SD conditions, the DTHs of the mutant lines were approximately 12 days later than that of SN265 (late season in Guangzhou, 113° E/23° N), whilst under LD conditions, the DTHs of the mutant lines were delayed by approximately 17 days compared to that of SN265 (Figure 1a,b).

**Figure 2:**
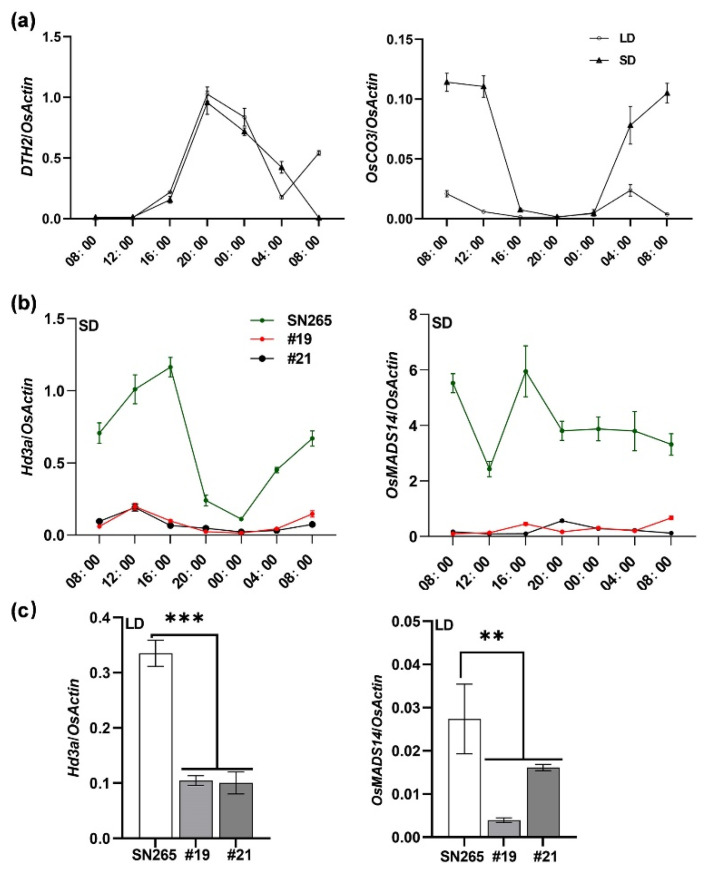
Expression analysis in SN265 and *dth2*-*osco3* lines via qRT-PCR under SD and LD conditions in Guangzhou. Under SD, leaves of 53-d-old plants were used for the expression analysis. Under LD, leaves of 80-d-old plants were used for the expression analysis. (**a**) expression of *DTH2* and *OsCO3* in SN265 under SD and LD, respectively. (**b**,**c**) expression of *Hd3a* and *OsMADS14* in SN265 and *dth2*-*osco3* lines under SD and LD, respectively. Error bars represent SD of three biological replicates. Values are means ± SD. **, *p* < 0.01. ***, *p* < 0.001.

To further explore how *DTH2* and *OsCO3* regulate heading in rice, we first examined the expression patterns of *DTH2* and *OsCO3* by qRT-PCR in SN265 grown in Guangzhou. Under SD conditions, both *DTH2* and *OsCO3* showed rhythmic expression patterns, whereas under LD conditions, only *DTH2* showed rhythmic expression patterns (Figure 2a). We further found that the transcript levels of *Hd3a*, a key flowering activator in rice [10], and its downstream effector *OsMADS14* [9,17] in #19 and #21 were significantly lower than those in SN265 under both SD and LD conditions (Figure 2b,c). Our results indicated that the disruption of both *DTH2* and *OsCO3* delays heading by down-regulating the *Hd3a*-*OsMADS14* pathway.

### 2.2. dth2-osco3 Increased Panicle Size and Grain Yield under SD Conditions

**Figure 3:**
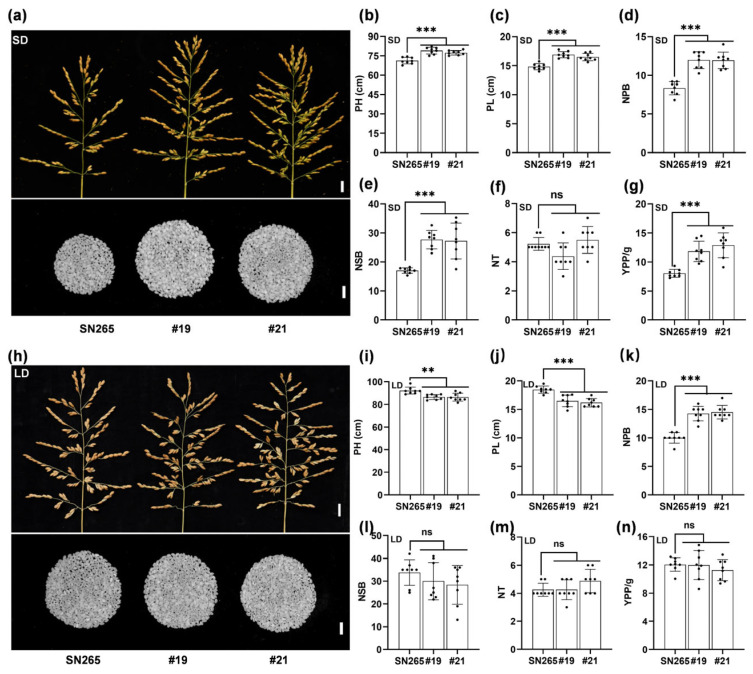
Double mutation of *DTH2* and *OsCO3* improves the agronomic performance of *japonica* rice under SD and LD conditions. (**a**) and (**h**) The panicle shape and number of polished grains of SN265 and *dth2*-*osco3* lines growing under SD and LD conditions. Scale bar for the panicles, 1 cm; scale bar for the polished grains, 10 mm. (**b**) and (**i**) the plant height, PH. (**c**) and (**j**) the panicle length, PL. (**d**) and (**k**) the number of primary branches, NPB. (**e**) and (**l**) the number of secondary branches, NSB. (**f**) and (**m**) the number of tillers, NT. (**g**) and (**n**) the yield per plant, YPP. YPP(**g**) = (the number of spikelets per plant × seed setting rate × per 1000-grain weight)/1000. Values are means ± SD. **, *p* < 0.01. ***, *p* < 0.001. ns, no significance.

To test the yield potential of the late-heading *dth2*-*osco3* mutants, some yield-related agronomic traits, including plant height (PH), panicle length (PL), the number of primary branches (NPB), the number of secondary branches (NSB), the number of tillers (NT), and yield per plant (YPP), were evaluated under both SD and LD conditions. Compared to SN265, the *dth2*-*osco3* lines (#19, #21) demonstrated a significant increase in PH, PL, NPB, NSB, and YPP but no difference in NT under SD conditions (Figure 3a–g). However, under LD conditions (early season in Guangzhou), the double mutant lines showed no significant differences in NSB, NT, and YPP along with reduced PH and PL, although there was still a significant increase in NPB (Figure 3h–n). These observations suggest that impaired *DTH2* and *OsCO3* improve the agronomic performance of SN265 in the *indica* rice-growing areas under SD conditions.

Taken together, our data suggest that *DTH2* and *OsCO3* negatively regulate the adaptation of agronomic traits, especially for DTH and YPP, in *japonica* rice growing at low-latitudes.

### 2.3. Haplotype Analysis of DTH2 and OsCO3

**Figure 4:**
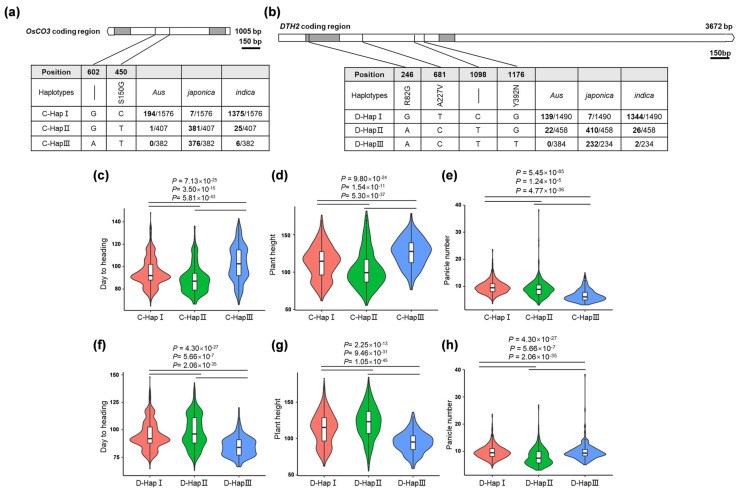
Haplotypes of *OsCO3* and *DTH2* in rice and their association with agronomic traits. (**a**) and (**b**) Haplotypes of *OsCO3* and *DTH2* and their distributions in *indica*, *japonica*, and *Aus* varieties. (**c**–**e**) The association between the haplotypes of *OsCO3* and days to heading, plant height, and panicle number. (**f**–**h**) The association between the haplotypes of *DTH2* and days to heading, plant height, and panicle number. The haplotype and agronomic trait data were retrieved on RFGB v2.0 (https://www.rmbreeding.cn/, accessed on 11 November 2022) and RiceVarMap v2.0 (http://ricevarmap.ncpgr.cn/, accessed on 11 November 2022).

To investigate the distribution of the haplotypes of *DTH2* and *OsCO3* in *japonica* and *indica* rice and validate their contribution to regional adaptation, we searched rice genomic databases for the functional nucleotide polymorphisms at the *DTH2* and *OsCO3* loci. We used RFGB v2.0 (https://www.rmbreeding.cn/, accessed on 11 November 2022) and RiceVarMap v2.0 (http://ricevarmap.ncpgr.cn/, accessed on 11 November 2022) [18,19,20] to identify the haplotypes of DTH2 and OsCO3 in 3000 rice germplasms. The data revealed that both *DTH2* and *OsCO3* had three major haplotypes, D-HapI~D-HapIII, and C-HapI~C-HapIII, respectively. D-HapI and C-HapI were mainly distributed in *indica* varieties, whereas most of the others were found in *japonica* varieties (Figure 4a,b). These three haplotypes of *OsCO3* and *DTH2* showed significant differences in yield-related traits such as heading date, plant height, and panicle number and may be associated with the phenotypic differentiation between *indica* and *japonica*. For example, C-HapIII-carrying varieties exhibited prolonged heading date, higher plant height, and reduced panicle number (Figure 4c–e), whereas the varieties with D-HapIII showed the opposite (Figure 4f–h). These data suggest that the adaptation of the aforementioned agronomic traits may be regulated by *OsCO3* and *DTH2* in *japonica* rice. We therefore postulate that the functional nucleotide polymorphisms at *DTH2* and *OsCO3* may serve as the target sites of genome editing in the “*indica* to *japonica*” projects.

## 3. Discussion

During a long-term domestication process, *japonica* and *indica* rice have evolved to be adapted to different environments [15]. This study demonstrated that the disruption of two heading-related genes, *DTH2* and *OsCO3*, greatly improved the agronomic performance of *japonica* cultivars growing in the *indica* rice cultivation areas, for example, in terms of heading date and yield per plant. The late-heading double mutant *dth2*-*osco3* was identified from a homozygous T_2_ population consisting of the genomic mutations of eight heading-related genes differentiated between *japonica* and *indica* rice. Indeed, a previous study has shown that *DTH2* has two functional nucleotide polymorphisms (FNPs) in *indica* rice IR24 and *japonica* rice Asominori, which plays an important role in the northward expansion of *japonica* [13]. We thus postulate that targeted editing of the genes differentiated between *japonica* and *indica* rice may help introduce beneficial mutations toward regional adaptation. In this regard, our work informs two critical loci regulating the adaptive traits of *japonica* varieties. When more and more such genomic loci are collected, especially for those contributing to the adaptation to temperature fluctuations and photoperiodic changes, we may eventually reach our final goal of the “*indica* to *japonica*” projects.

## 4. Materials and Methods

### 4.1. Plant Materials and Plant Growth Conditions

Rice (*Oryza sativa* L. ssp. *japonica*) Shennong265 (SN265) was obtained from Dr. Liang Tang’s lab at Shenyang Agricultural University. All the experiments were performed on an experimental farm at South China Agricultural University in Guangzhou (GZ, 113° E/23° N), China, during the normal rice-growing early season (12–13.5 h day length from mid-March to July) and the late season (11.5–13.5 h day length from mid-July to November) (Figure S2).

### 4.2. Genetic Transformation

We designed eight sgRNAs specifically targeting the coding regions of *DTH2*, *OsCO3*, *OsLF1*, *SDG725*, *OsGS1*, *OsDof12*, *OsNF-YC2*, and *OsPUP7* using CRISPR/Cas9 systems [16]. The sgRNAs were cloned into the binary vector pYLCRISPR/Cas9P_ubi_-H2, carrying the Cas9 gene and an sgRNA expression module for editing. The primer design and vector construction were following the CRISPR-GE (http://skl.scau.edu.cn/, accessed on 20 July 2019, Guangzhou, China) (Table S1) [16]. The vectors were introduced into SN265 via the Agrobacterium-mediated transformation approach. The targeted genomic regions of the eight genes were examined by PCR using the primers listed in Table S2. The sequencing results were decoded by the Degenerate Sequence Decoding (DSD) method [21].

### 4.3. Trait Evaluation and Data Analysis

Eight plants in the middle of each row for SN265 and the *dth2*-*osco3* lines were analyzed individually to score PL, NPB, NSB, NT, and YPP after ripening. Thirty-five plants were analyzed individually to score PH and DTH. DTH was scored as time (in d) from seed-soaking to the emergence of the first panicles. PH was measured as the distance from the ground surface to the tip of the tallest panicle, and PL is the distance from the panicle base to the uppermost spikelet. NPB and NSB were measured as described previously [22]. NT refers to the effective tiller number. YPP (g) = (the number of spikelets per plant × seed setting rate × per 1000-grain weight)/1000.

### 4.4. RNA Extraction and Gene Expression Analysis

Sixty-day-old plants grown in the early season and the late season were used for the expression analysis of the flowering-time genes. Leaves were harvested every 4 h within 1 day (7-time-points). Total RNAs were extracted using an Eastep^®^Super Total RNA Extraction Kit (Promega, Shanghai, China) and reverse-transcribed into cDNA using a Transcript One-Step gDNA Removal and cDNA Synthesis SuperMix (Transgen, Beijing, China) kit. Quantitative reverse transcription polymerase chain reaction (qRT-PCR) assays were performed using a PerfecStart Green qPCR SuperMix (Transgen, Beijing, China) kit on a C1000 CFX96 Real-time PCR Detection System (Bio-rad, Hercules, CA, USA) with the following setting: 95 °C for 2 min, 42 cycles of two-step amplification (95 °C for 30 s, 60 °C for 30 s). PCR was repeated three times for technical repetition. The primers used for gene expression analysis are listed in Table S3.

## 5. Conclusions

In this study, we found that the double mutations of *DTH2* and *OsCO3* (*dth2*-*osco3*) significantly delayed heading under both SD and LD conditions in Guangzhou and manifested great yield increase under SD conditions. We further demonstrated that the heading-related *Hd3a*-*OsMADS14* pathway was down-regulated in the *dth2*-*osco3* mutant lines. Our findings provide new resources for the “*indica* to *japonica*” projects in South China.

## Data Availability

Data are contained within the article.

## Supplementary Materials

The supporting information can be downloaded at: https://www.mdpi.com/article/10.3390/ijms24087346/s1.
