# Supplementary material for: Double Mutation of Days to Heading 2 and CONSTANS 3 Improves Agronomic Performance of Japonica Rice under Short Daylight Conditions in Southern China
Source: Int J Mol Sci. 2023 Apr 16;24(8):7346. doi: 10.3390/ijms24087346 (PMC10138775; doi:10.3390/ijms24087346)
Supplement: Supplementary file 1 [file ijms-24-07346-s001.zip › ijms-2317725-supplementary.pdf]

Supplementary materials

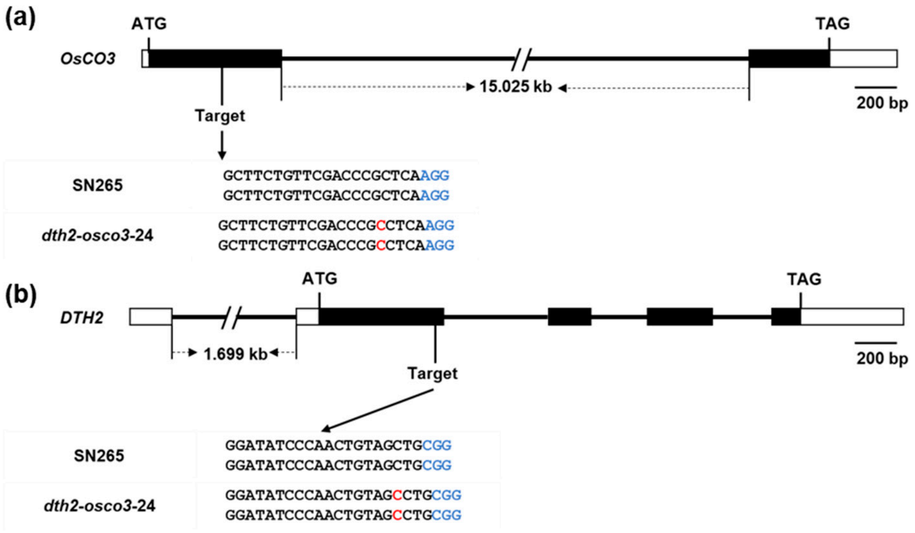

Figure S1. Schematic diagram and the position of the editing target site of the *OsCO3* and *DTH2*. Black boxes and lines in between represent exons and introns, respectively. The target sequence is shown in black and the PAM sequence (NGG) in blue. Underneath the target sequence is the sequence alignment to show the 1-bp insertion (highlighted in red) induced by gene editing in an independent transgenic line (*dth2-osco3*-24) (a and b).

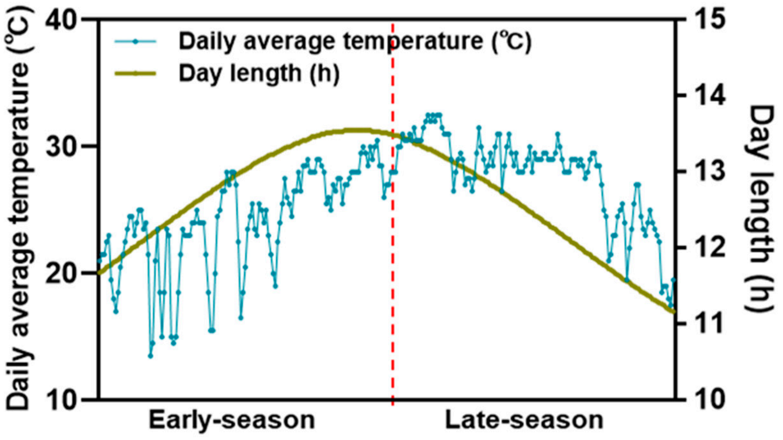

Figure S2. Relationship between day-length and the daily average temperature in two seasons.

Table S1. Amplification primers for expression cassettes.

Primer Names	Sequences (5′-3′)
DTH2-gRT1	GATATCCCAACTGTAGCTGGTTTCAGAGCTAGAAAT
DTH2-OsU6bT1	CAGCTACAGTTGGGATATCCAACACAAGCGGCAGC
OsCO3-gRT1	GCTTCTGTTCGACCCGCTCAGTTTCAGAGCTAGAAAT
OsCO3-OsU3T1	TGAGCGGGTCGAACAGAAGCTGCCACGGATCATCTGC
OsLFL1-gRT1	TACAGCGACGTAAGCCAGCTGTTTCAGAGCTAGAAAT
OsLFL1-OsU6aT1	AGCTGGCTTACGTCGCTGTACGGCAGCCAAGCCAGCA
SDG725-gRT1	CTGTACCACCAGCACCTGCTGTTTCAGAGCTAGAAAT
SDG725-OsU6aT1	AGCAGGTGCTGGTGGTACAGCGGCAGCCAAGCCAGCA
OsGS1-gRT1	AGAGTACACCCTCCTCCAGAGTTTCAGAGCTAGAAAT
OsGS1-OsU6bT1	TCTGGAGGAGGGTGTACTCTCAACACAAGCGGCAGC
OsDof12-gRT1	ACAAGATCCTGCCGTGCCCCGTTTCAGAGCTAGAAAT
OsDof12-OsU6cT1	GGGGCACGGCAGGATCTTGTCTGAGCCTCAGCGCAG
OsNF-YC2-gRT1	GCGTGCGAGCTCTTCATCCGTTTCAGAGCTAGAAAT
OsNF-YC2-OsU6cT1	GGATGAAGAGCTCGCACGCCTGAGCCTCAGCGCAG
OsPUP7-gRT1	TCGTCATCGGCGCCCTGATGGTTTCAGAGCTAGAAAT
OsPUP7-OsU3T1	CATCAGGGCGCCGATGACGATGCCACGGATCATCTGC
DTH2-gRT1	GATATCCCAACTGTAGCTGGTTTCAGAGCTAGAAAT

Table S2. Detection primers for target sites mutation.

Primer Names	Sequences (5′-3′)
NGS-*DTH2*-F	CTCGGAGTGATCGCACGGCAGACCATAAACTGTTAC
NGS-*DTH2*-R	CTGAGAGGCTGGATGGGCTGTGCTAGCTATATCCAAC
NGS-*OsCO3*-F	CTCGGAGTGATCGCACGTGTCAACTCCTCCTGGCCA
NGS-*OsCO3*-R	CTGAGAGGCTGGATGGTCGACGACGCGTCCTCCTTCT
NGS-*OsLFL1*-F	CTCGGAGTGATCGCACGGGCAGATTTATTCGAACGT
NGS-*OsLFL1*-R	CTGAGAGGCTGGATGGGGTGTACTGAAGCAAATACCC
NGS-*SDG725*-F	CTCGGAGTGATCGCACCCTGCTGCTGAACTTGTATTC
NGS-*SDG725*-R	CTGAGAGGCTGGATGGGGCAGGATGGAAATCAATACC
NGS-*OsGS1*-F	CTCGGAGTGATCGCACGTCACAGCCATCTCAGCATAA
NGS-*OsGS1*-R	CTGAGAGGCTGGATGGAAGCCACAATCAAACAGAC
NGS-*OsDof12*-F	CTCGGAGTGATCGCACAGAGGTCGTCGACACCGAGGA
NGS-*OsDof12*-R	CTGAGAGGCTGGATGGTCCAGTACCTCTGGCAGTTC
NGS-*OsNF-YC2*-F	CTCGGAGTGATCGCACTGGCGCGGATCAAGAAGATCAT
NGS-*OsNF-YC2*-R	CTGAGAGGCTGGATGGCGACGAGGAAGTCGAACACGTC
NGS-*OsPUP7*-F	CTCGGAGTGATCGCACTGGTGACGCTGGTGCAGTC
NGS-*OsPUP7*-R	CTGAGAGGCTGGATGGTGAACGCGAGCTGCGTCGACA

Table S3. Primers for qRT-PCR reaction.

Primer Names	Sequences (5′-3′)
qRT-*DTH2*-F	GAGATGGGATGAATCTTCTGC
qRT-*DTH2*-R	GTCTCCATATACGCTCCCATCA
qRT-*OsCO3*-F	CAATTTCAGGCGCGGGTACA
qRT-*OsCO3*-R	GAGGTGGTGATGTCTGGCAC
qRT-*Hd3a*-F	GTCTACCCCTAGCTAACGATGA
qRT-*Hd3a*-R	CACCATCATATATATGTTGTGTGTCG
qRT-*OsMADS14*-F	CGGTTGCGAGACGAGGAA
qRT-*OsMADS14*-R	GAAAGACGGTGCTGGACGAA
Actin1-3A	ACCACAGGTAGCAATAGGTA
Actin1-5B	CACATTCCAGCAGATGTGG
